# What Can Be Learned from the Partitioning Behavior of Proteins in Aqueous Two-Phase Systems?

**DOI:** 10.3390/ijms25126339

**Published:** 2024-06-07

**Authors:** Vladimir N. Uversky, Pedro P. Madeira, Boris Y. Zaslavsky

**Affiliations:** 1 Department of Molecular Medicine, Byrd Alzheimer’s Research Institute, Morsani College of Medicine, University of South Florida, Tampa, FL 33612, USA; vuversky@usf.edu; 2 Centro de Investigacao em Materiais Ceramicos e Compositos, Department of Chemistry, 3810-193 Aveiro, Portugal; p.madeira@ua.pt; 3 Cleveland Diagnostics, 3615 Superior Ave., Cleveland, OH 44114, USA

**Keywords:** aqueous two-phase system, partitioning, structural signature, protein, protein–protein interaction, aggregation

## Abstract

This review covers the analytical applications of protein partitioning in aqueous two-phase systems (ATPSs). We review the advancements in the analytical application of protein partitioning in ATPSs that have been achieved over the last two decades. Multiple examples of different applications, such as the quality control of recombinant proteins, analysis of protein misfolding, characterization of structural changes as small as a single-point mutation, conformational changes upon binding of different ligands, detection of protein–protein interactions, and analysis of structurally different isoforms of a protein are presented. The new approach to discovering new drugs for a known target (e.g., a receptor) is described when one or more previous drugs are already available with well-characterized biological efficacy profiles.

## 1. Introduction

Proteins are crucial biological machines responsible for the vast majority of cellular functions. They exist in multiple forms, being single- or multi-domain, monomeric or oligomeric, soluble or membrane-bound, and ordered or intrinsically disordered. Ordered proteins might contain different proportions and arrangements of α-helical and β-structural elements [1,2,3]. In their turn, intrinsically disordered proteins can be disordered to different degrees and are known to contain variable levels of partial structure [4]. Proteins are almost always engaged in a multitude of interactions, many of functional importance. They virtually never act alone, being interconnected with various partners, and their engagement in crucial protein–protein interactions results in the formation of complex networks. Proteins can undergo a wide spectrum of posttranslational modifications (i.e., covalent attachment of various chemical groups to the different amino acid residues after the completion of protein biosynthesis) [5,6,7]. The biological activities of proteins are precisely tuned and controlled by multiple factors. The normal state of action of a proteome known as a protein homeostasis, or proteostasis, is supported by a set of cellular activities that maintain the health of the proteome and the organism [8,9,10]. Distortions in proteostasis are linked to protein misfolding and are associated with various cardiovascular, metabolic, neurodegenerative, and oncological diseases, as well as aging [8,11,12,13,14,15,16,17,18,19]. It was also indicated that many proteins engaged in the pathogenesis of various diseases are intrinsically disordered [20].

Because of their vital importance for most physiological and pathological processes, proteins have attracted a lot of attention from researchers. For example, proteins are essential components of cells and are responsible for carrying out various functions based on the instructions encoded in genes. Their ability to tightly and specifically bind to other molecules allows them to perform diverse functions. One well-known role of proteins is serving as enzymes, which accelerate specific chemical reactions. Enzymes are involved in most metabolic reactions and play a role in DNA processes such as replication, repair, and transcription.

Additionally, proteins are involved in cell signaling and can transmit signals between cells. Some proteins, like insulin, function outside the cell to transmit signals to other tissues. Membrane proteins act as receptors, binding signaling molecules and triggering biochemical responses in the cell. Specific proteins, known as structural proteins, provide rigidity to biological components, while motor proteins like myosin, kinesin, and dynein generate mechanical forces. Not surprisingly, multiple experimental and computational tools, approaches, and methods have been developed for protein structural and functional analysis. Experimentally, protein structures can be analyzed at the residue level by high-resolution techniques, such as X-ray crystallography, NMR, and cryo-electron microscopy (cryo-EM). X-ray crystallographic analysis is the oldest of these three approaches. It continues to serve as a major source of information on protein structure, but the use of protein NMR spectroscopy and cryo-EM is also gaining momentum. However, these modern methods for protein structure determination are not widely available. Furthermore, the time and cost of sample preparation and the cost of operation and use of the NMR, cryo-EM or X-ray crystallography facilities, combined with the need for highly trained specialists for conducting the corresponding experiments and interpreting the results, made them prohibited for many researchers. The use of these three techniques is limited by the accessibility of the corresponding instrumentation, the utilization of which requires specialized expertise. Alternatively, a very broad spectrum of low-resolution techniques is utilized to gain knowledge on different structural aspects of these complex biological machines. The physical principles of these approaches range from spectroscopic to hydrodynamic to thermodynamic and although they also need specialized equipment and expertise, these techniques are more accessible to researchers. Since proteins are biopolymers with complex structural organization, it is recognized that the successful and accurate description of their structural properties relies on the multiparametric approach, which includes different techniques sensitive to different levels of proteins’ structural organization, the idea equally applicable to ordered and intrinsically disordered proteins [21,22,23]. 

This review represents a rather different approach based on the analysis of the peculiarities of protein partitioning in aqueous two-phase systems (ATPSs). Such systems are formed in aqueous mixtures of two (or more) polymers or a single polymer and salt or organic compound once the concentrations of phase-forming compounds or temperature exceed a certain threshold, leading to the separation of the originally homogeneous solution into two (or more) aqueous phases, as extracted in [24,25,26]. 

These systems have found a wide range of applications in biotechnology [27], as the extraction, purification, isolation, cleanup, and recovery of various biological compounds, including proteins, can be achieved by ATPS-utilizing approaches based on liquid–liquid phase separation [24,25,26,27,28,29,30,31,32,33,34,35,36,37,38,39,40,41,42,43,44,45,46,47,48,49,50,51,52]. The aqueous two-phase extraction utilized in protein separation is based on the fact that various proteins distribute differently between the two phases. 

In addition to the ATPSs formed in aqueous mixtures of two (or more) polymers or a single polymer and salt, which are known to be used in analytical purposes (see below), at certain temperatures and surfactant concentrations, aqueous solutions of surfactants can form ATPSs as well. Here, under specific conditions, homogeneous aqueous micellar solution separates into two aqueous phases characterized by different concentrations and sizes of micelles [53,54,55,56,57,58,59,60,61]. Although such surfactant-based ATPSs have been successfully utilized for the separation of viral particles and proteins [53,54,55,56,57,58,59,60,61], they have not been used in analytical purposes as of yet.

Another group of ATPSs includes systems formed by a single polymer and surfactant, such as PEG-Triton X-100 or dextran-octylglucoside [62]. Such ATPSs have been used for the fractionation of membrane proteins prior to analysis by mass spectrometry [63], but the best of our knowledge, these systems have not been utilized in analytical purposes as of yet. Additionally, some osmolytes (such as glycine, betaine, and choline) have been reported to form ATPSs in mixtures with PEG or potassium phosphate, and these ATPSs have been used for protein partitioning [64]. 

It is important to emphasize here that a defining feature that allows the classification of a given system as an ATPS is the fact that although the phases are immiscible and differ in their solvent properties, each of the phases contains well over 80% water on a molal basis. Therefore, although two-phase systems can be formed by water, (hydrophobic) ionic liquids, and aqueous solutions of inorganic salts [65,66,67,68,69,70,71,72,73], or by ionic liquids and polymers [74,75,76,77], ionic liquids and surfactants [78,79], and water and water-miscible organic solvent (e.g., ethanol) with inorganic salt additives [80,81,82,83,84], such two-phase systems cannot be classified as ATPSs, as, strictly speaking, in these cases, both resulting phases cannot be considered aqueous.

The distribution of proteins between phases depends on the ATPS composition and peculiarities of the protein amino acid sequence and 3D structure. These observations indicate that the peculiarities of the partition behavior of a protein in various ATPSs is a reflection of the peculiarities of its tertiary structure. Therefore, the partition of a protein in ATPSs can be used to generate its structural signature, which is sensitive to single-point mutation, conformational changes upon binding of different ligands, the detection of protein–protein interactions, protein misfolding and aggregation, and the presence and analysis of structurally different isoforms. This article reports various applications of this technique and provides multiple illustrations of its usefulness for very different analyses. 

## 2. Determination of Partition Coefficient

**Figure 1:**
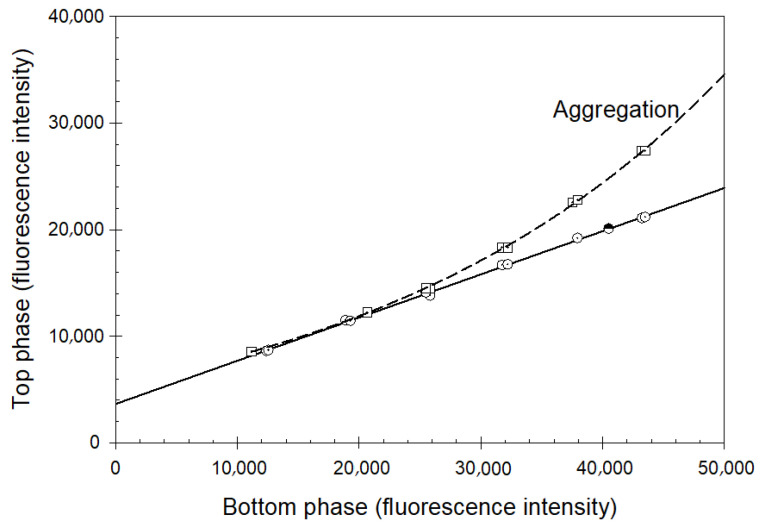
The concentration of a protein in the top phase is plotted against the concentration of the protein in the bottom phase (concentration is represented by the analytical signal, in this case, fluorescence in the o-phthalaldehyde (OPA) assay). Deviation of the plotted curve from the linearity indicates protein aggregation. Data for this plot are taken from [85]. Circles represent the partitioning of monomeric protein, while squares represent the partitioning of aggregated protein (refer to the text for details). White symbols represent data from known samples. The black and white circle is from a “blind” sample, confirming it is not in the aggregated form.

The partition behavior of proteins in an ATPS is characterized by the value of the partition coefficient, *K*, which is defined as the ratio of the protein concentrations in the top to the bottom phases. For analytical applications, the desirable conditions must meet the following two requirements: (a) the protein under study should partition within a reliably measurable range (0.1 < *K* < 10) (under conditions of extreme partitioning, the range of concentrations of the protein in the protein-poor phase is the range of low and unreliable analytical signals); and (b) the partition coefficient *K* values should be sensitive to changes in interest (such as a change in the protein conformation, structural modification, etc.). A detailed description of the partition coefficient measurements for analytical purposes was reported in the literature [85]. Figure 1 represents an illustration of the process of the partition coefficient determination for a query protein.

Once the protein concentrations (or analytical signals with intensity proportional to the protein concentration) in the phases are measured, the partition coefficient of the protein can be determined. The protein concentrations (analytical signals) determined in the top phases are plotted against those determined in the bottom phases. A typical resulting plot is presented in Figure 1. The observed linear dependence is described as:

(1) $$C_{Top}^{i}=a+b\times C_{Bottom}^{i}$$

where *C^i^* is the protein concentration (analytical signal) measured in a particular phase denoted by the subscripts “*Top*” and “*Bottom*”, and *a* and *b* are constants. The coefficient *a* is related to the interference of the phase components with the analytical signal and may be close to 0. The coefficient *b* value represents the protein partition coefficient *K*.

## 3. Peculiarities of Partitioning Can Be Used for Protein Quality Control

**Figure 2:**
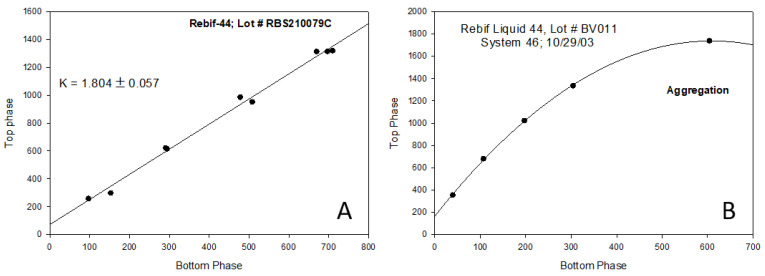
The effect of aggregation on Rebif’s partition behavior. The plots were built using data reported by L. Mikheeva et al. [86]. Figure (**A**) shows the partitioning of a non-aggregated Rebif formulation, while Figure (**B**) shows an aggregated one.

The linearity of the plot in Figure 1 indicates that the protein partition coefficient K is independent of the protein concentration. This implies that identical species are distributed between the coexisting phases within the protein concentration range used in the experiment. On the other hand, deviation of the experimentally determined relationship between the *C^i^_Top_* and *C^i^_Bottom_* from linearity indicates that changes in the protein concentration result in the appearance of species with partition behavior different from that present at lower protein concentrations, generally implying the formation of protein aggregates or oligomers, or even oligomer dissociation. In other words, the partition behavior of a single monomeric protein—or an oligomeric one, like hemoglobin—is represented by a straight line. When a sample contains monomers or oligomer units and oligomerizes/aggregates, or when oligomers dissociate, the overall partition coefficient may deviate from linearity because the individual partition coefficients of these different components are different. It should be noted that the deviation from linearity may be leading to increased or decreased partition coefficient values depending on the particular properties of the new species in the solution. This idea is illustrated by Figure 2 showing the effect of aggregation on the partition behavior of recombinant β-interferon (Rebif) formulations. The analyzed samples, provided by Serono, were part of a feasibility study conducted by Analiza, Inc., to verify the applicability of structural signature technology for the detection of protein aggregation in pharmaceutical formulations. 

**Figure 3:**
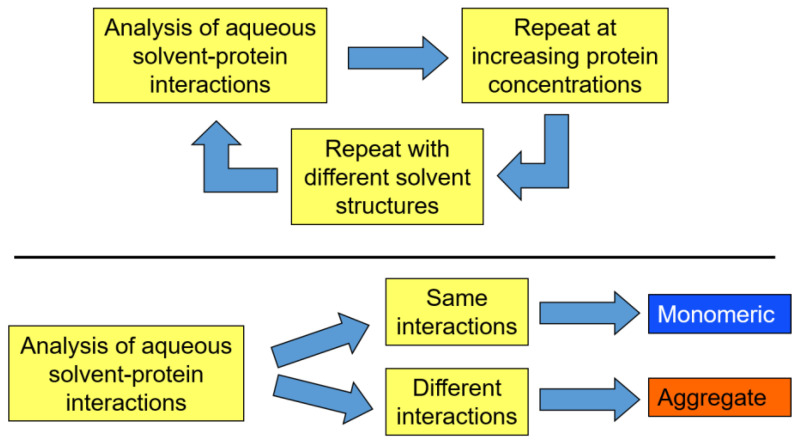
Schematic representation of the use of the structural signature technology in the analysis of protein aggregation.

These considerations indicate that the nonlinearity of a plot representing the partition behavior of a query protein (see Figure 1 and Figure 2) serves as a reflection of the presence of protein aggregation. It is clear that this technique can be used for quality control during recombinant protein production. Since the cost of isolation represents the largest contribution to the cost of recombinant protein production, and since misfolded protein, which often tends to aggregate, should not be purified, ATPS-based quality control similar to that shown in Figure 1 and Figure 2 may be implemented during manufacturing. The standard ATPS protocol for the characterization of the individual proteins in the presence of contaminants was reported in the literature [85]. Figure 3 schematically represents a protocol of the utilization of structural signature technology in the analysis of protein aggregation. 

## 4. Effect of Environmental Factors on Protein Partitioning in ATPSs

Protein distribution in ATPSs must depend on the protein environment as well as on the presence of other proteins or other solutes. As an example, for individual purified human albumin, the partition coefficient *K* in a given ATPS is 0.69 ± 0.01, whereas in a similar ATPS, the value is 1.28 ± 0.02 in blood (plasma/serum). For human transferrin, the corresponding values are 1.78 ± 0.04 and 0.53 ± 0.12. For human immunoglobulin alone or in blood (plasma/serum), we observed *K* values of 4.42 ± 0.02 and 4.19 ± 0.04, while for prostate-specific antigen the values were 1.90 ± 0.06 and 1.11 ± 0.01.

**Figure 4:**
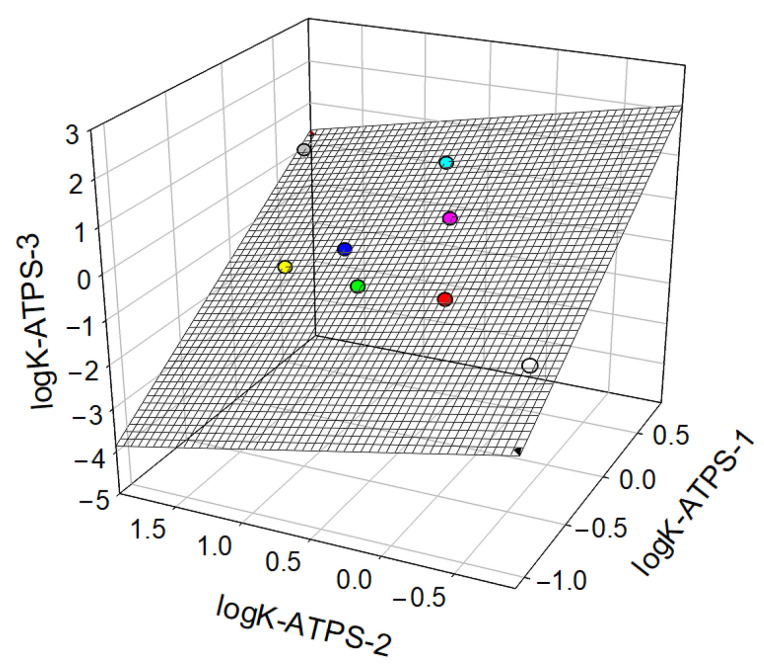
The interrelationship between the logarithms of partition coefficients for proteins in dextran-75–PEG-8000–0.8 M CsCl–0.01 M NaPB, logarithms of partition coefficients for the same proteins in PEG-8000–0.33 M NaCl–0.1 M UB, and logarithms of partition coefficients for the same proteins in PEG-600–0.4 M NaSCN–0.17 M K/NaPB ATPSs. Plot was built using data reported by L.A. Ferreira et al. [89]. The colors refer to different proteins.

The partition coefficients of various proteins in ATPSs of various ionic and polymer compositions are typically linearly interrelated [87,88], as illustrated in Figure 4. 

**Figure 5:**
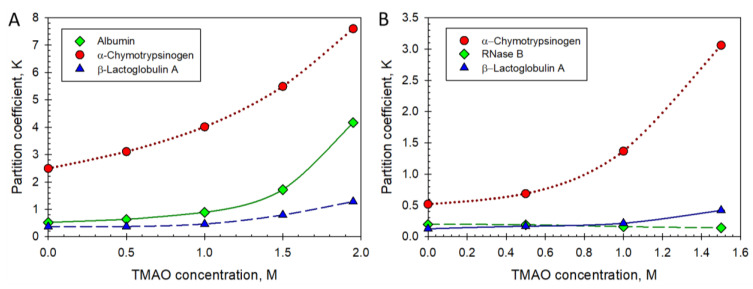
(**A**) Partition coefficients of albumin, β-lactoglobulin A, and α-chymotrypsinogen A in Dextran-75-PEG-600 ATPS as functions of trimethylamine N-oxide (TMAO) concentration. (**B**) Partition coefficients of α-chymotrypsinogen A, ribonuclease B, and β-lactoglobulin A in Ficoll-70-PEG-8000 ATPS as functions of trimethylamine N-oxide (TMAO) concentration. Plot was built using data reported in the literature [90].

The commonly used salt additives may strongly influence the partition behavior of proteins. Adding certain nonionic additives may influence the behavior of some proteins even more [90], as illustrated graphically in Figure 5. 

The data reported by L.A. Ferreira et al. [90] show that the partition behavior of small organic compounds and proteins may be manipulated in ATPSs of various polymer and ionic compositions using nonionic organic compounds. The relative preference of a protein, such as albumin or α-chymotrypsinogen, for a given phase may be changed drastically by increasing the concentration of trimethylamine N-oxide (TMAO) additive. Since in the polymer–polymer ATPSs of various polymer and ionic compositions, the partition behavior of both, small organic compounds and proteins may be manipulated by adding a small nonionic organic compound, TMAO, one can conclude that a solute’s molecular weight is not crucial for its partition behavior in the polymer–polymer ATPSs, and that the partition behavior of proteins and small organic compounds is instead governed by the solute–water interactions in the coexisting phases [90]. In this context, trimethylamine N-oxide (TMAO) realizes its effect on the solute partition behavior via its influence on the solvent features of water in the coexisting phases of ATPSs [91,92].

## 5. Partitioning in ATPSs as a Tool for Deriving Unique Protein Structural Signatures

Comprehensive analysis of the partition of proteins in various ATPSs can be used for extracting important information on the protein structure in the form of a structural signature. In fact, the structural signature represents an effective combination of physicochemical information about protein–solvent interactions under various conditions and a follow-up mathematical treatment that condenses the information into a signature. A signature is best described as a fingerprint of the structure. In a completely analogous manner to the way actual fingerprinting is used, the structural signature is simply a convenient means to represent complex structural information of relevance. A signature could be a numerical value, a visual picture, or any other means to convey the quantitative information obtained from the experiments. It has a direct one-to-one correspondence with the underlying information base (the entire structural state of the biomolecule). The key features of the signature are:

- It has a direct one-to-one correspondence with the underlying information base (the entire structural state of the biomolecule). For example, two separate signatures could be developed for lots typical of nominal biological efficacy and for lots known to have reduced (or lack of) biological efficacy;
- It is easier/faster/less expensive to retrieve the structural signature than to determine the actual structure.

The construction of a signature comprises two steps: application-unique experimental conditions provide multiple degrees of sensitivity toward specific structural changes, and mathematical tools integrate these data into numerical and visual information, rapidly and automatically. For example, while using this technique in the comparative analysis of the mutants of a given protein, partition coefficients for a wild-type protein and a protein mutant are measured in three or more different ATPSs. The Euclidean distance between the set of logarithms of partition coefficients for the mutant and the wild-type protein is calculated and represents the structural signature.

Once examples of samples with known biological efficacy levels or examples with known degrees of structural difference are used to develop a set of representative signatures, the process of quality control testing comprises (rapidly and easily) obtaining the signature of the unknown product and then assessing its similarity to other signatures of known samples. Assessing the similarity is performed visually or mathematically using one or more algorithms. 

The experimental portion of obtaining the signature is founded upon examination of the biomolecule interactions with specialized aqueous-based solvent media. The protein–solvent interactions are examined using the well-known technique of aqueous two-phase partitioning (ATPP). 

Under the context of a signature, different ATPSs are used to probe (be sensitive to) structural aspects that differ among products of nominal or reduced activities. While the information from one experiment may only respond to certain changes, the construction of multiple systems that respond to the different aspects of the structure provides the means to combine the resultant information and present it as a signature of the structure.

**Figure 6:**
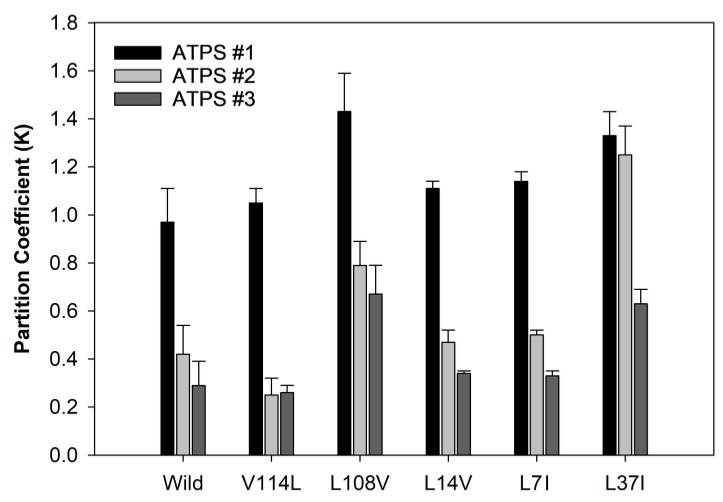
Distribution of various mutants of Staphylococcal nuclease in three different aqueous two-phase systems. The colors identify the systems used in the analysis. Plot was built using data reported by L. Mikheeva et al. [86].

The significant finding reported by K. Berggren et al. [93] demonstrated that the nature and 3D arrangement of the solvent-exposed groups of amino acids define the protein partition behavior. The difference between globular and intrinsically disordered proteins (IDPs) [94] is that residues in a globular protein might be buried inside a hydrophobic core or be located on the protein surface and exposed to the surrounding solvent, whereas in the intrinsically disordered proteins or regions, most of the amino acid residues are solvent-exposed. In line with these observations, it was shown that the peculiarities of the partitioning of proteins in a set of ATPSs of different ionic compositions can be used to quantify structural differences between an IDP, α-synuclein, its variants, and globular proteins [95]. Furthermore, the partition behavior of a given protein can be noticeably affected by single-point mutations [27,95,96]. For example, the replacement of tryptophan-143 for phenylalanine in a small globular protein, interleukin-7, was shown to lead to ca. 3-fold increases in the partition coefficient, from 0.262 ± 0.003 to 0.745 ± 0.004. In our study, various mutants of Staphylococcal nuclease in different ATPS systems were characterized by noticeable differences in their partition behavior, as illustrated by Figure 6.

These examples clearly show that the peculiarities of the partition behavior can indicate the characteristics of a protein, or its structural signature, that can be affected by its environment and amino acid substitutions.

The described behavior of proteins under various partition conditions indicates that the improved description of the protein 3D structure and conformation may be obtained using a partition in several different systems. Functions of ordered globular proteins are related to their unique 3D structures, which are commonly determined by X-ray crystallography and NMR spectroscopy [97]. However, explicit structural information at atomic resolution is often unnecessary, especially in cases such as the classification of isoforms with slightly different primary or secondary structures, posttranslational modifications, or conformation [27,96,98]. 

These and other applications would ideally be approached with a simple analytical technique, which provides a condensed index (vector or even a single number) of the structure conveniently for comparative purposes and would also have the required sensitivity and specificity to delineate structural changes in interest. We developed such a method [99] capable of distinguishing closely related structural forms of a protein without detailed structural analysis. We showed how highly complex and detailed higher-order structural information can be condensed into a useful numerical index. The present comparative approach is based on quantifying the interactions of a protein with aqueous media with different solvent properties. 

To design the appropriate partition conditions for a given series of proteins, it is necessary to perform two rounds of screening. The first or preliminary screening aims to select ATPSs that, for a given protein, would provide the partition coefficient within the robust analytical range (typically 0.1–10). For this purpose, a single protein sample is needed. These experiments will typically consume about 2–5 mg of protein, depending on the sensitivity of the assay employed for the evaluation of the protein concentration. Once the ATPSs for this first screening step are selected, the second step is needed. This step must establish conditions providing sufficient differences between the *K* values for the proteins tested. This screening typically uses a limited number of pre-selected ATPSs with different salt compositions, and the amounts of the proteins required at this stage are usually below 1 mg for each protein (again, depending on the assay employed for the concentration assay). Once the suitable ATPSs are selected, all the protein samples may be analyzed. 

It was shown in [99] how sensitive conformational information can be obtained using a signature and how different states of the structure of biomolecules can be compared against a reference state. The study also demonstrated how to condense the difference between the signatures of a different conformation into a simple numerical value. Further, several representative applications for such condensed information sets that reflect differences between signatures were disclosed.

As an illustration of the power of this approach, it was shown in [27] that conformational changes induced by different cations [99] in human apo-transferrin are readily detected with the ATPS extraction technique. The corresponding data are listed in Table 1.

The data presented in Table 1 indicate clearly that there is a difference in the partitioning of apo-transferrin and transferrins saturated with different metal ions. The differences observed are due to different conformations of transferrins induced by the binding of different metals. The data given in Table 1 also show that the changes in the partition coefficient value of a protein induced by the binding of a partner depend on the particular two-phase system employed, although the changes are observed in all the systems employed.

The data reported in Table 1 represent a structural signature corresponding to each ligand. These data can be visually displayed in several ways. Sometimes, it is convenient to reduce the signature’s complexity further. One way to accomplish signature condensation is to calculate a (normalized) Euclidian distance for each signature versus a reference case. These distances are calculated using the logarithms of the partition coefficient. In the following example, we chose to describe the distance between each conformational state corresponding to each ligand against that of the apo-transferrin signature. One formula for calculating such a distance is:

(2) $$d_{i,j}=\sqrt{\sum_{k=1}^{n}{\left(\frac{c_{j,k}}{\max\left(c_{k}\right)}-\frac{c_{i,k}}{max(c_{k)}}\right)}^{2}}$$

where the distance is calculated between any signature and the reference signature *i* for *n* aqueous systems. The distance data reported in Table 1 could be interpreted individually for each ligand as a measure of the overall similarity between the conformation induced by that ligand to that of the free receptor. Other data transformation and condensation methods could be readily devised, depending on the ultimate use of the signature. Thus, for example, if one wishes to rapidly compare the similarity of one ligand-induced conformation to another or versus a reference state, which might be that of a ligand whose biological activity is known, then the distance measure is one convenient way to express the similarity compactly. Another application could be using the similarity distance to assess how close the signature of a particular isoform or a modified form of a biomolecule (e.g., with a single-point mutation) is to that of the intact biomolecule. Another possibility using the distance measure of signature similarity is to conveniently compare many microheterogeneous proteins (e.g., glycoproteins) produced using recombinant DNA techniques in non-mammalian host cells. In this case, the signature of each lot representing the average conformation state of a mixture could be readily compared against that obtained from a well-characterized lot with a known biological activity level.

We examined [100] several batches of human, porcine, and bovine insulins from different manufacturers in six ATPSs. As an illustration, the data obtained for human insulin are listed in Table 2. 

As seen from Table 2, the subset of three ATPSs is sufficient for obtaining a reliable signature of the protein structure. However, for ribonucleases A and B, a reliable difference was observed [100] using only two different ATPSs. For the A and B isoforms of β-lactoglobulin from cow milk, differing by two amino acid residues out of the 162 residues—β-lactoglobulin A has Asp-residue in position 64 and Val-residue in position 118, while β-lactoglobulin B has Gly-residue in position 64 and Ala-residue in position 118—the required subset of different ATPSs was larger than for the ribonucleases, and four ATPSs were used (see Table 3) [100].

The study [100] was the first to show how to develop a compact representation of the protein structure, a signature that could be used to compare several proteins and confer sufficient specificity to the technique. While the structural distance cannot delineate any arbitrary and specific structural differences between the two samples, it has the following three important benefits: (i) it could be derived to detect and classify very subtle and specific changes in the structure of interest; (ii) it is useful for a range of practical applications in biotechnology and medicine; and (iii) it can be measured using automated high-throughput protocols with readily available robotic liquid handlers and other conventional laboratory instruments. 

These properties make the method particularly useful for current analytical needs in biotechnology, including the comparability of biosimilars [97], stability testing of immunotherapeutics, and even field testing of counterfeit biologics—in recognition that each manufacturer could have a unique structural signature with a pedigree going back to its proprietary cell line. 

**Figure 7:**
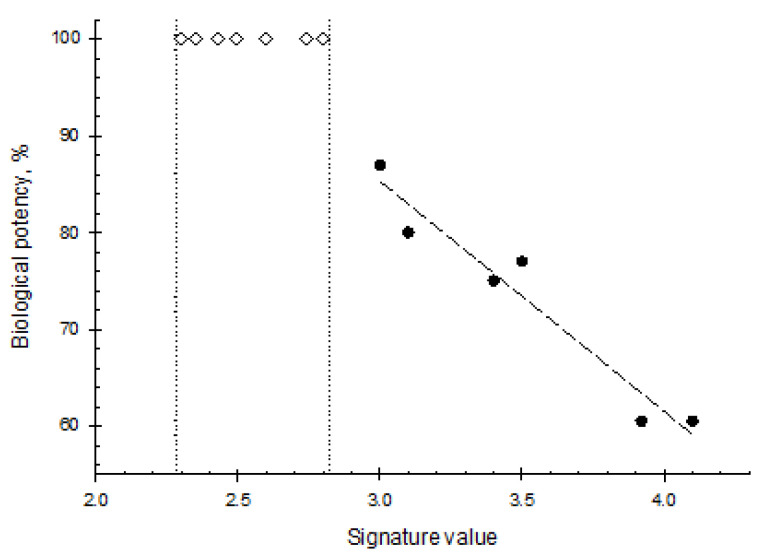
The biological potency of different batches of human growth hormones plotted against their behavior in various aqueous two-phase systems. The diamond symbols show the range of signature values for different batches of the product with 100% potency. Plot was built using data reported elsewhere [101].

Our efforts [101] to use the peculiarities of ATPS partition as a quality control and a rapid test of the lot-to-lot consistency, product stability, in-process control, etc., are illustrated graphically in Figure 7, showing the corresponding results for the recombinant human growth hormone. 

**Figure 8:**
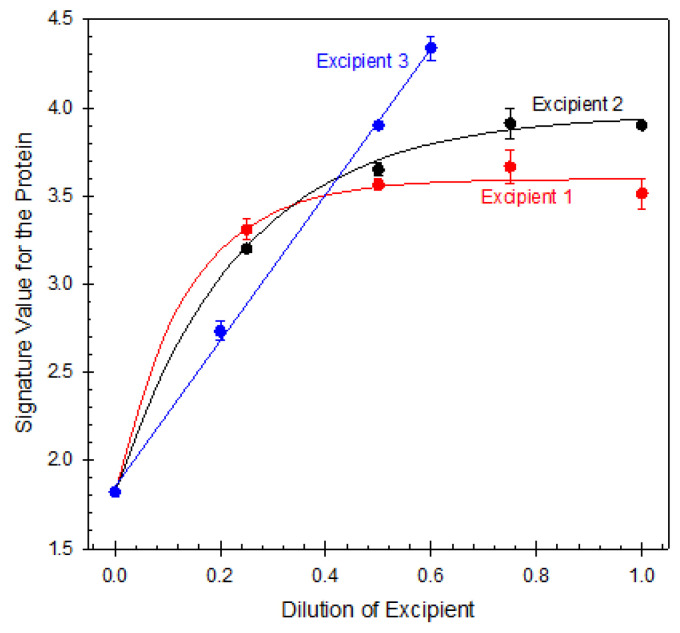
Effect of excipients on protein X conformation as revealed by it partition in different ATPSs. Plot was built using data reported by L. Mikheeva et al. [86].

Similarly, the results of studying double-blind samples of various formulations of a protein X from one of the top five pharmaceutical companies are partially illustrated in Figure 8. The data show that not only do various excipients affect the protein conformation quite differently, but also that all excipients affect the 3D structure of this protein.

**Figure 9:**
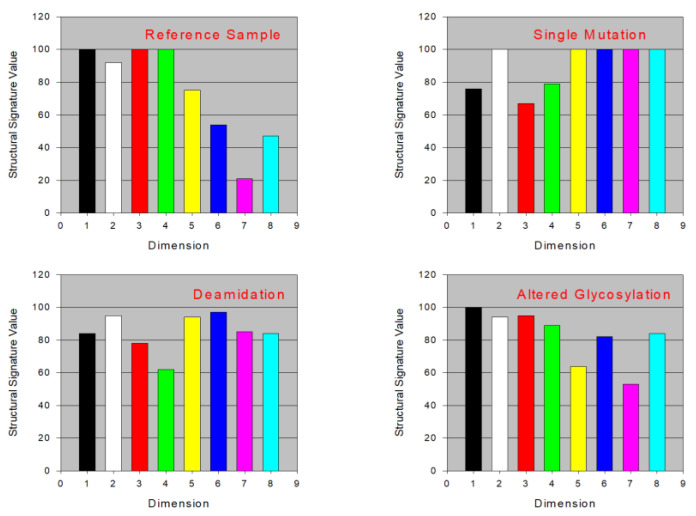
Effect of different treatments on the structural signatures of several proteins. *X*-axis indicates the number of the ATPS, with each “dimension” representing a different ATPS. Therefore each color represents a distinct structural signature. Plots were built using data reported elsewhere [86].

A comparison of different protein samples subjected to the specific treatments is illustrated graphically in Figure 9. It is seen that the structural properties of the proteins, being presented in a form of structural signature value determined from the analyses of these proteins in ATPSs, are undoubtedly affected by single mutations, deamidation, and glycosylation. The data reported in Figure 9 clearly demonstrate that ATPS-derived structural signatures represent a useful approach for the detection of various structural alterations of proteins.

## 6. Partitioning in ATPSs as a Tool for the Analysis of Structurally Different Isoforms of a Query Protein

Proteins and glycoproteins often exist in vivo as structurally different isoforms. The relative amounts of these isoforms are often changed in pathological processes. Analyses of the relative amounts of the isoforms of certain marker proteins are important in the diagnosis, prognosis, and monitoring of diseases and response to treatment. Carbohydrate-deficient transferrin (CDT) [102], an isoform of transferrin, provides one illustrative example. Changes in the structure of the carbohydrate part of transferrin, such that the sialic acid content is decreased, are recognized to be indicative of alcohol abuse. The ratio of CDT to total transferrin, indicative of alcohol abuse, refers to the relative amount of the subpopulation of modified transferrin with low sialic acid content concerning the total amount of all transferrin isoforms [103] (see below). 

A second example of subpopulations in a mixture [104] is the ratio of glycated hemoglobin to total hemoglobin content, which is an important indicator of the long-term status of diabetes [105]. The partitioning of mixtures of human hemoglobin and human glycated hemoglobin in different aqueous two-phase systems is related to the ratio of the amounts of proteins in the mix (see Table 4).

Yet another ratio of isoforms that is of clinical importance, being associated with an increased risk of thrombosis, is that of the blood coagulation factor V (FV) existing as a mixture in two forms with different thrombogenic potential, FV1 and FV2 [106]. Measurements of the concentrations of such isoforms are usually performed using complex analytical techniques that require discrete steps of separation (chromatographic or electrophoretic) of the diagnostically relevant protein isoforms before assaying their concentrations. An analytical method that detects the ratios in question without first separating the protein isoforms should be more efficient than separation-based procedures. 

Earlier, we reported an approach for evaluating the ratio of two structurally different compounds in a mixture that does not require any preparation steps [103]. This approach is based on using the ATPS partition coefficient calculated as the ratio of analytical signals measured in the two phases and proportional to the corresponding concentrations of the compound [103]. 

The total amount of the compound partitioned in ATPSs may be expressed as a sum of the compound concentrations multiplied by the volume of each phase. The overall partition coefficient of a mixture of two compounds (mixture of the two isoforms of a given protein or a mixture of two proteins), *K*_Σ_, may be defined as the ratio of the overall concentrations of these compounds in the two phases as follows:

(3) $$K_{\Sigma }=\frac{C_{1T}+C_{2T}}{C_{1B}+C_{2B}},$$

where *C*_1*T*_, *C*_1*B*_, *C*_2*T*_, and *C*_2*B*_ are concentrations of the compounds 1 and 2 in the top and bottom phases, respectively, with the total concentrations of these compounds *C*_1_ and *C*_2_ in the system being *C*_1_ = (*C*_1*T*_ + *C*_1*B*_) and *C*_2_ = (*C*_2*T*_ + *C*_2*B*_). The ratio of the one-compound concentration to the overall concentration of both compounds, *R*, can be evaluated as:

(4) $$R=\frac{C_{1}}{C_{1}+C_{2}}$$

It was shown in [103] that combining the corresponding equations and eliminating the concentration variables leads to the dependence of the overall partition coefficient of the compound mixture, *K*_Σ_, on the ratio of the compounds, *R*:

(5) $$K_{\Sigma }=\;\frac{A+B\times R}{C+D\times R},$$

**Figure 10:**
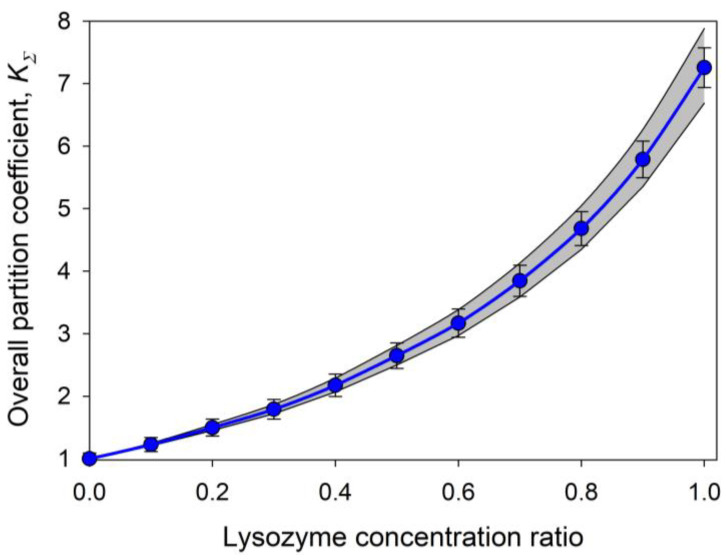
Relationships between the overall partition coefficient, *K*_Σ_, for mixtures of hen egg white lysozyme and human hemoglobin and the content ratio of lysozyme, *R_lysozyme_*, defined by Equation (4). Experiments were conducted in the PEG-600-phosphate buffer, pH 6.5 ATPS. The blue circles with error bars show the experimental data points. The solid blue line represents the theoretical curve calculated using Equation (5). The gray shaded area reflects the 95% confidence interval around the theoretical curve. Plots were built using data reported by Zaslavsky et al. [103].

The *A*, *B*, *C*, and *D* parameters in this equation are defined in reference [103]. The capability of this approach to predict the partition behavior of mixtures of the different compounds was analyzed by Zaslavsky et al. [103]. As one of the examples of the power of this approach, the mixture of the two globular proteins with noticeably different partition coefficients, lysozyme with an individual *K_HEL_* value of 7.29 ± 0.31 and hemoglobin with an individual *K_Hb_* value of 0.96 ± 0.02, was examined [103]. Figure 10 represents the comparison of the experimental data and theoretical relationship for this system and shows that within the experimental error bars, the experimental and calculated data match, thereby emphasizing the validity of such an analysis [103]. 

**Figure 11:**
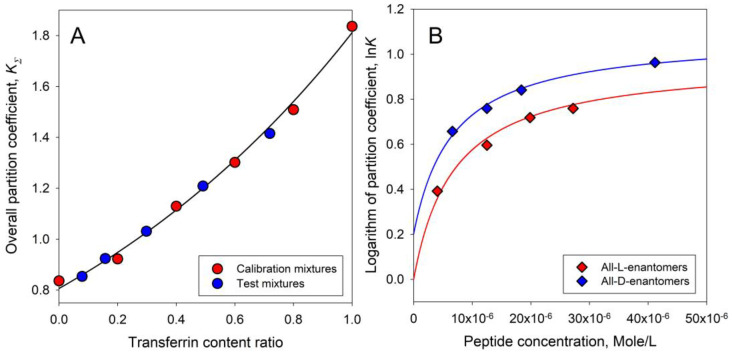
(**A**). Relationships between the overall partition coefficient K_Σ_ for mixtures of human transferrin and carbohydrate-deficient transferrin (CDT) in the aqueous PEG-600-phosphate buffer, pH 6.95, two-phase system, and the transferrin content ratio. Red circles represent the data for the calibration mixtures; blue circles represent the test mixtures. (**B**). The logarithm of partition coefficient, ln*K*, values for all-L- and all-D-enantiomers of Cecropin A(1-13)-Melittin(1-13) (red and blue diamonds, respectively) as a function of the peptide concentration in the aqueous Dex–PEG two-phase system containing 0.15 M NaCl in 0.01 M sodium phosphate buffer, pH 7.3. Plots were built using data reported in the literature [103].

In another example, the comparison between the predicted and experimentally observed distribution behavior of intact human transferrin and its carbohydrate-deficient isoform is illustrated in Figure 11A. In yet another illustrative example, the behavior of mixtures of L- and B-enantiomers of the hybrid peptides in the ratios of 1:3, 3:1, and 1:1 was examined [103] to test the assumption of the formation of mixed aggregates. The experimental data for all the mixtures are plotted in Figure 11B together with those predicted above. The results imply that the aggregation of the peptides increases with their concentrations, and that L- and D-enantiomers form aggregates that are characterized by the significant differences in their partition behavior.

## 7. Partitioning in ATPSs and Protein Interactions

**Figure 12:**
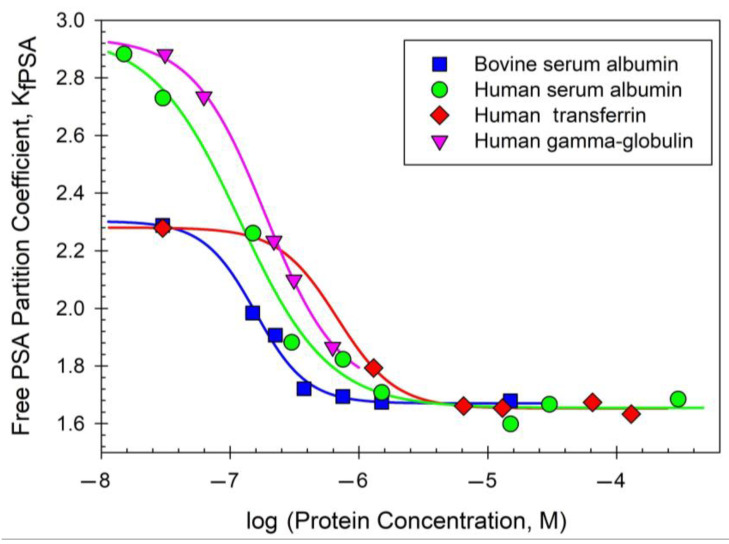
The partition coefficient of free PSA (*K_fPSA_*) as a function of the indicated protein concentration. The plot was built using data reported by O. Fedotoff et al. [108].

An attempt in [107] to explore the structural features of the proteins determining their behavior in different ATPSs, however, failed as expected, implying our lack of comprehension of protein structure in solution. Obviously, our current level of understanding of the effects of protein–protein interactions on the partition behavior of query proteins is even lower than the knowledge of the individual proteins’ structures. This is illustrated by the analysis of the effect of different human proteins, such as albumin, IgG, and transferrin, on the partition of prostate-specific antigen (PSA) in ATPSs [108]. This analysis revealed that all the examined proteins affected PSA partition behavior, as illustrated in Figure 12.

The association of PSA with human serum albumin and γ-globulin (immunoglobulin G, IgG) was reported in the literature [107]. The data obtained here are insufficient to conclude whether PSA binds to the proteins examined or not. We analyzed the partitioning of the proteins in the ATPS employed, and their partition coefficient values are: *K_BSA_* = 0.205 ± 0.006; *K_HSA_* = 0.254 ± 0.006; *KTf* = 0.586 ± 0.007; and *KIgG* = 5.6 ± 0.19. The partition coefficient of the free PSA alone is *K_fPSA_* = 1.90 ± 0.06. Therefore, the partition coefficients of the free PSA alone and in mixtures with the proteins examined are significantly different from those of the individual proteins. These data show that while both albumins and transferrin distribute predominantly into the lower phase of the ATPS, γ-globulin/IgG mostly distributes into the upper phase of the system used. The properties of a protein-PSA complex are hard to predict but it seems counterintuitive (though possible) that the PSA complex with γ-globulin (if formed) distributes with a partition coefficient significantly lower than the ones observed for individual partners (free PSA alone and γ-globulin). It also seems difficult to explain that complexes of PSA with proteins as different as albumin, transferrin, and γ-globulin would display essentially identical partition behavior. Hence, it is assumed that the presence of a protein induces conformational changes in the PSA molecule and plays a role in changes in the PSA partition coefficient. This assumption is consistent with the view in [109,110,111,112,113,114,115] that the macromolecular environment may cause changes in the protein conformation. Another possibility may be that since the PSA is a serine protease, it might interact with different proteins to find if there are peptide bonds to cleave. This question remains open.

Protein–protein interactions have been studied numerous times using the ATPS technique. The theory of the ligand effect on the partition behavior of a protein was described by Brooks et al. [116] and further developed by Cordes et al. [117] and Suh and Arnold [118]. Numerous examples of studies of protein–protein and polynucleotide–polynucleotide interactions using the ATPS partition technique are presented in the literature [119,120]. The complex formation of spectrins with calmodulin [121,122] is an example of protein–protein interactions studied in detail by aqueous two-phase partitioning. 

**Figure 13:**
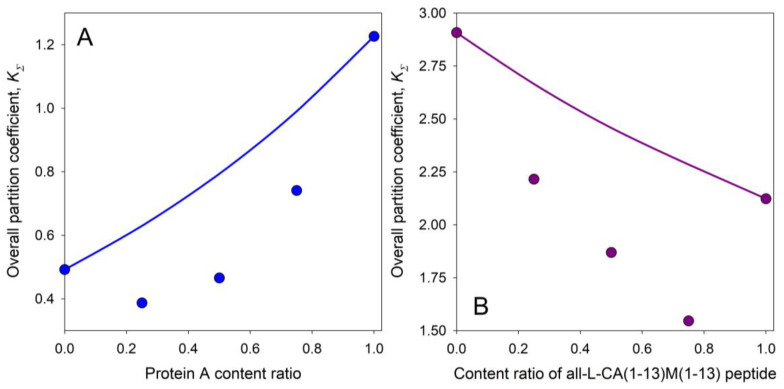
(**A**) Overall calculated and experimental distribution behavior of the mixtures of protein A and human IgG in the aqueous Dex-Ficoll two-phase system containing 0.15 M NaCl in 0.01 M sodium phosphate buffer, pH 7.3. The solid line represents the $K_{\Sigma }$ values calculated under the assumption of the lack of protein–protein interactions. The circles denote the experimental points. (**B**) The overall calculated and experimental $K_{\Sigma }$ values for mixtures of all-L- and all-D-enantiomers of Cecropin A(1-13)-Melittin(1-13) in the ratios of 1:3, 1:1, and 3:1 as a function of the ratio of peptide concentrations in the mixture in the aqueous Dex–PEG two-phase system containing 0.15 M NaCl in 0.01 M sodium phosphate buffer, pH 7.3. The solid line represents the K_Σ_ values calculated with the assumption of the lack of peptide–peptide interactions. The circles represent the experimental data points. Plots were built using data reported elsewhere [103].

The approach suggested here may also be used for detecting protein–protein interactions. Deviation of experimentally determined KS values for the mixture of two proteins from those predicted using theoretical treatment described elsewhere [103] would imply that the compounds in the mixture under study do not partition independently but rather interact to form a complex that is partitioned differently from the two constituent proteins. This is illustrated in Figure 13A by the results of the study of mixtures of protein A with human IgG and in Figure 13B for mixtures of all-L- and all-D-enantiomers of Cecropin A(1-13)-Melittin(1-13).

In collaboration with several pharmaceutical companies, we examined the binding of different receptors with multiple drugs and drug candidates of various classes. We found that the described approach was successful in differentiating between different classes of these compounds. 

**Figure 14:**
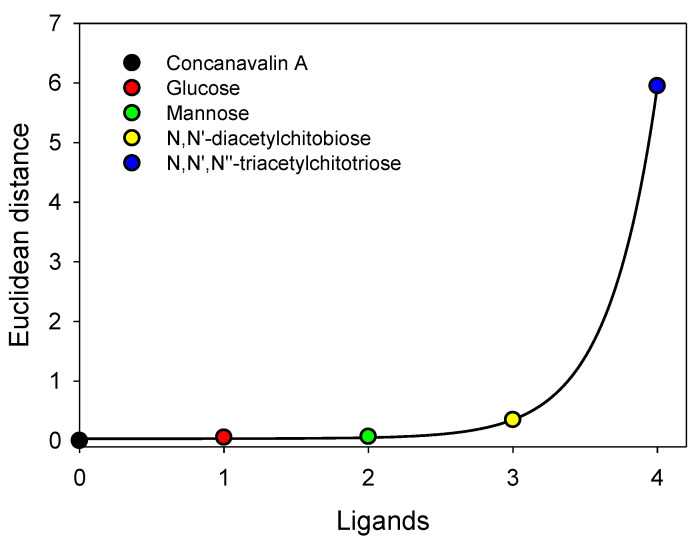
Euclidean distances for the 1:1 complexes of lysozyme with carbohydrates (data for concanavalin A were used as a reference point). Plot was built using data reported elsewhere [30].

Protein interactions with nonionic compounds may also induce unexpected conformational changes. The partition behavior of a protein under given conditions is determined by the nature and steric arrangement of solvent-exposed residues and, therefore, changes in partition behavior may be interpreted as changes in the protein conformation. Therefore, changes in the partition behavior associated with the complex formation reflect binding-induced conformational changes. As an example, the data listed in [27] are illustrated graphically in Figure 14. The Euclidean distances were calculated as described in the literature [99] using the set of *K* values for concanavalin A as the reference point. N,N′,N″-triacetylchitotriose and N,N′-diacetylchitobiose bind to the active site of lysozyme, while glucose and mannose do not. The fact that the nonspecific weak binding does not significantly affect the partition behavior of a protein is reflected in the fact that in the presence of glucose and mannose, the Euclidean distances in Figure 14 are close to zero. On the other hand, the data obtained for N,N′,N″-triacetylchitotriose and N,N′-diacetylchitobiose indicate that these carbohydrates induce certain conformational changes in the protein. 

Therefore, based on the peculiarities of the partition in the ATPS, the signature of a structural state of a biomolecule can be created. The signature created can be shown numerically and visually. The binding of different ligands induced different conformational changes, which were then monitored to obtain signatures. This is illustrated by an example of the characterization of the interaction of β-Lactoglobulin from bovine milk with its partners. The partition coefficients for the examined mixtures of β-lactoglobulin and its binding partners [99] are presented in Table 5.

Analysis of the data presented in Table 5 indicates that the partition coefficient *K* values for the protein changes in the presence of each ligand examined in almost all the systems (except in the presence of bromethanol in the Dex-PEG system). However, inadequate conclusions might have been drawn if only one system had been used to analyze the conformational changes induced by the ligand binding. Retinol acetate binding increases the protein’s *K* value in the PEG-Phosphate, Dex-PEG, and Dex-Ficoll-NaSCN systems. It reduces the *K* value in the Dex-Ficol system employed. At the same time, the binding of nitrophenyl phosphate and bromethanol decreases the protein *K* value in all the systems used here.

The use of a series of different aqueous two-phase systems significantly improves our ability to describe the conformational changes induced in a protein by the binding of different ligands. The various systems to be used include those formed by a single polymer and inorganic salt(s), by different pairs of polymers, and by the same pair of polymers and different salt additives. 

**Figure 15:**
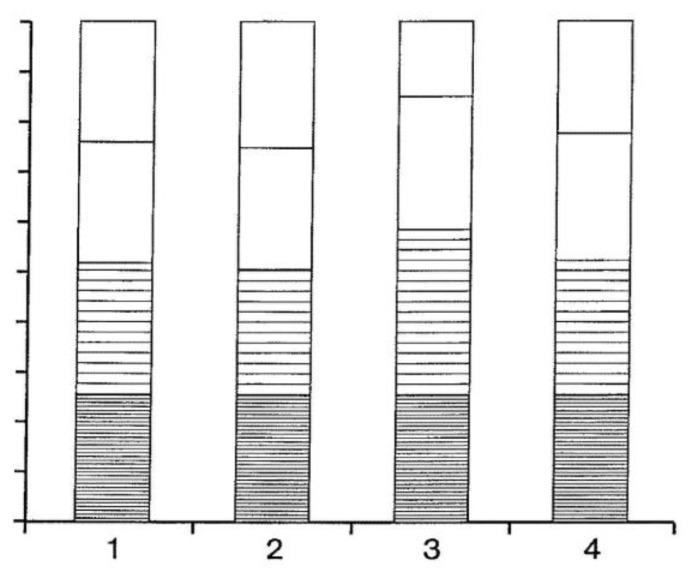
A visual representation of the signatures, using normalized bar graphs, whose numerical values are listed in Table 5. The values 1–4 on the abscissa refer to PEG-phosphate, DEX-Ficoll, Dex-Ficoll-NaSCN, and Dex-PEG systems, respectively. The height of each bar is equal, and the relative contribution of each system to each bar height is denoted by its vertical extent. Also, the signature of each structural state is understood as the pattern obtained for the four cases (shown on the abscissa) by the assembly of bars, wherein the height of each Sub-section corresponding to the normalized K value at a different aqueous system.

The signature of the state of the structure can be expressed in various ways. For example, a visual representation of the information in Table 5 can be constructed by first normalizing the *K* values obtained for each system by the largest value, then displaying the resulting matrix in graphical form. This was carried out, and the results are shown in Figure 15. In this particular graphical representation, the height of each bar is equal, and the relative contribution of each system to each bar’s height is denoted by its vertical extent. Also, the signature of each structural state is understood as the pattern obtained for the four cases (shown on the abscissa) by the assembly of bars, wherein the height of each sub-section corresponds to the normalized K value in a different aqueous system.

It was also shown in [99] how to use a structural signature to discern between specific and nonspecific binding of ligands to a biomolecule by observing the structural signatures of the conformational state induced by such binding. Table 6 displays the partition coefficients, K, for hen egg white lysozyme and its mixtures with different binding partners in the indicated ATPSs. The data was previously published [102].

The binding of nonspecific ligands, such as glucose and mannose, changes the partition coefficient that can be distinguished from the binding of specific ligands. Specifically, the protein partition coefficient value changes did not exceed 5%. In comparison, the binding of specific ligands at the active site of lysozyme resulted in partition coefficient changes ranging from 6% up to 80% depending on the particular aqueous two-phase system. Further, the example demonstrates that the sets of partition data, like those in Table 2, and the analysis of the patterns of partition behavior of protein–ligand complexes in a set of different two-phase systems provide information of greater reliability and quality than one would expect from a collection of single two-phase signatures.

For example, evaluating a one-dimensional signature represented by the partition coefficient in the Dex-Ficol system does not clarify the types of conformational modifications induced by each ligand. However, this can be achieved by using the entire information set as a multi-dimensional signature using the normalized values of the partition coefficient in a radar plot.

In this particular representation of the signature, nonspecific binding events were recognized as those that do not result in significant visual (or numerical) deviations from the signature of the free receptor alone, while the three ligands that are known to have an increased degree of specificity (from N-Acetyl-D-glucoseamine to N,N’-Diacetylchitobiose to N,N’,N”-Triacetylchitotriose, respectively) produced a signature that increasingly deviates from that of the free receptor. However, by using the graphical representations of the signature, it was also easy to determine that the component of the signature did not provide valuable information to provide a useful signature. For a signature to be useful, it must provide one-to-one correspondence with the underlying information, even if the basis on which the underlying information gives rise to the signature is unknown. Thus, if we plot, e.g., the variation of the components of all the signatures for each system separately, it becomes evident that the last two systems (Dex-Ficoll-NaSCN and Dex-PEG) are less sound for discerning structural differences amongst the different ligands.

Importantly, the conformational signature can be used to assess the biological activity of an unknown drug, using signatures of known drugs as reference cases [99].

Sometimes, when discovering new drugs for a known target (e.g., a receptor), one or more previous drugs are already available with well-characterized biological efficacy profiles. Assuming that the biological activity of a receptor is reflected in its conformational state, then the problem is how to use the conformational information already available for previous drugs (ligands) for the same receptor to rapidly evaluate the anticipated biological activity of a new drug candidate. More generally, the question is how to predict the profile of a new drug candidate’s conformationally related bioactivities if such profiles exist for other drugs. Examples of conformationally related bioactivities are biological activity level and toxicity, expressed as a consequence of undesired binding to other receptors that produce conformational states that activate undesired biological activities. It is of significant value for analyzing many classes of receptors that undergo multiple conformational changes in response to bindings to different drugs. For example, many transcription factors, e.g., estrogen receptors, exhibit numerous conformational states in response to binding to different estrogens. The discovery of new estrogens that modulate the estrogen receptors of great current interest since it is widely recognized that the intermediate conformations are of practical interest for discovering new estrogens that exhibit favorable biological activity profile in various tissues in the body. Thus, instead of merely turning on the receptor upon binding, many drugs could be tailored to induce specific conformational states that could improve bioefficacy. In the following hypothetical example, we explore, using arbitrary numerical values, several methods that could be used to classify or predict the biological activity of an unknown drug for the same receptor. The techniques to experimentally obtain the numerical values underlying the signature were extensively demonstrated and discussed above.

Mathematically, the signature for each drug is a vector comprising n values, each corresponding to a particular system. It is assumed that the signature corresponds to the conformational state and that the conformational state is related to the biological activity of the receptor. If the bioactivity of the receptor, when bound to a specific drug, is known, and could be classified numerically, e.g., using a normalized scale in comparison with other drugs by assigning 0 for pure antagonist and 10 for pure agonist, the bioactivity could be mathematically related to the signature. The mathematical techniques used to relate the signature(s) to the bioactivity/activities are generally referred to as mapping. These could be linear or nonlinear, could involve simple concepts such as distance measures (like the measures shown for previous examples), are either local or global in their range of validity, etc. The map is essentially a mathematical relationship between the input, represented by a vector of values, and the output, represented (typically) by a single value.

If only one signature and one biological activity measure are available, corresponding to one known drug, then a simple comparison of the two signatures (known and unknown drugs) is, in general, not sufficient to predict the bioactivity of the unknown drug. One exception is when the two signatures are very similar, thus producing the same biological effect. If two drugs of known and different levels of bioactivities are available, then the signature for the unknown drug could be compared using, e.g., distance measurements to the two known signatures. The ratio between the two distances could be used as a ratio of the predicted bioactivity to both known activities. For example, if the three (normalized) signatures and their respective activities are as shown in Table 7 below:

Then, using Equation (2), the distance of the signature of Z to the two known drugs, X and Y is: Z–X: 0.79; Z–Y: 0.53. Therefore, the bioactivity of Z is expected to be closer to that of Y by a factor of 0.79/0.53 = 1.5, and a simple calculation using that ratio shows the predicted activity to be 4.

In a more general case, there will be multiple signatures and multiple bioactivity levels, but the same methodology could be followed. Other mapping alternatives, including matrix techniques, such as singular value decomposition, least squares, etc., could offer powerful and general tools. For many signatures available for the same receptor, other mapping and prediction options can be used. Nonlinear regression, artificial neural networks, etc., could be used to construct the mapping. Once the mapping is known, it could be used with the signature vector of the unknown drug to predict its activity. The latter case is of specific value for modern drug discovery, in which the biological activity of many potential drugs is routinely measured, e.g., in cell-based assays or using animal models. Only a few drug candidates (typically one) are eventually tested in clinical trials, but the previously available biological activity levels for many ligands that were not promoted to clinical trials could be used to develop the mapping of usefulness for predicting the activity of unknown drugs.

It is also possible to develop multiple signatures for several receptors of the same drug. Thus, it is possible to select the desired biological profile of a drug for multiple biological targets, using the methodologies described herein. Finally, it is also possible to derive multiple mappings between a given signature and multiple biological responses to predict the responses to an unknown drug, using the techniques described herein. For example, estrogenic compounds are known to produce a biological profile comprising different activity levels in several tissues in the body. The design of SERM compounds (Selective Estrogen Receptor Modulators) attempts to optimize the biological profile, and the present methodology could be used for this or similar applications by considering several mappings of the same signature to different bioactivity levels.

## Figures and Tables

**Table 1:** Partition coefficients of apo-transferrin and its complexes with different cations in various ATPSs (data from [99]).

Protein	ATPS 1	ATPS 2	ATPS 3	ATPS 4	Distance, d_io_
Apo-transferrin	1.07 ± 0.02	1.31 ± 0.03	1.23 ± 0.01	0.47 ± 0.01	0
Fe^3+^-transferrin	0.66 ± 0.004	1.13 ± 0.01	1.152 ± 0.004	0.51 ± 0.02	0.43 ± 0.02
Al^3+^-transferrin	0.69 ± 0.001	1.038 ± 0.003	0.97 ± 0.01	0.528 ± 0.001	0.48 ± 0.02
Cu^2+^-transferrin	0.72 ± 0.02	1.10 ± 0.01	1.075 ± 0.003	0.537 ± 0.002	0.41 ± 0.02
Bi^3+^-transferrin	0.75 ± 0.01	1.121 ± 0.001	1.056 ± 0.002	0.514 ± 0.002	0.37 ± 0.001

ATPS 1: Dextran-64–Ficoll-400–0.01 M NaPB, pH 8.6. ATPS 2: Dextran-64–Ficoll-400–0.25 M Na_2_SO_4_–0.01 M NaPB, pH 8.6. ATPS 3: Dextran-64–Ficoll-400–0.25 M Li_2_SO_4_–0.01 M NaPB, pH 8.6. ATPS 4: Dextran-64–Ficoll-400–0.13 M CsCl–0.01 M NaPB, pH 8.6.

**Table 2:** Structural (Euclidean) distances calculated from distribution coefficients for human insulin samples in various ATPSs (data from [100]).

Vendor	Lot#	ATPS ^a^				
		1–6	1–5	1–4	1–3	1–2
MP Biomed	3188F	0.152	0.152	0.150	0.126	0.126
Sigma	123K1243	0.125	0.122	0.121	0.097	0.087
Sigma	081K13562	0.122	0.122	0.122	0.079	0.079
Sigma	123K8416	0.014	0.014	0.014	0.008	0.008
Calbiochem	B52891	0.079	0.070	0.067	0.055	0.038

^a^ Distance for each sample calculated from the *K* values in the indicated number of ATPSs.

**Table 3:** Partition coefficient values for β-lactoglobulin A and β-lactoglobulin B from bovine milk in different aqueous two-phase systems.

ATPS	β-Lactoglobulin A	β-Lactoglobulin B
PEG-600-NaPB	3.596 ± 0.010	1.840 ± 0.003
Dex-PEG-NaCl-NaPB	0.048 ± 0.005	0.067 ± 0.003
Dex-PEG-NaPB	0.097 ± 0.011	0.123 ± 0.007
Dex-PEG-Li_2_SO_4_-NaPB	0.748 ± 0.015	0.404 ± 0.011
Dex-PEG-NaSCN-NaPB	0.096 ± 0.002	0.167 ± 0.013

**Table 4:** Partition coefficients for Glycohemoglobin Control-N (contains 7.6% GHb) and Glycohemoglobin Control-E (contains 15.0% GHb) in various ATPSs *.

ATPS	Control-N	Control-E
I	0.573 ± 0.027	0.634 ± 0.019
II	0.337 ± 0.021	0.269 ± 0.009
III	0.205 ± 0.020	0.290 ± 0.015
IV	0.836 ± 0.015	1.113 ± 0.029
V	1.025 ± 0.018	1.277 ± 0.0110

* ATPS: I—Dextran-500-PVP(K-15) − 0.15 M NaCl − 0.15 M NaPB, pH 4.9; II—Dextran-500 − PEG-8000 − 0.15 M NaPB, pH 4.9; III—Dextran-500 − PEG-8000 − 0.15 M NaPB, pH 6.5; IV—PEG-600 − 21.0% K/NaPB, pH 6.7; V—PEG-1000 − 16% K/NaPB, pH 7.6.

**Table 5:** Partition coefficients, *K*, of bovine β-lactoglobulin with different binding partners in the indicated ATPSs (protein/binding partner molecular ratio 1:1 in each case).

Binding Partner	ATPS (K Value)			
	I	II	III	IV
-	1.796 ± 0.005	0.836 ± 0.003	0.451 ± 0.002	0.180 ± 0.008
Retinol acetate	1.858 ± 0.016	0.812 ± 0.006	0.468 ± 0.002	0.196 ± 0.001
Nitrophenol phosphate	1.714 ± 0.012	0.823 ± 0.018	0.420 ± 0.001	0.103 ± 0.001
Bromoethanol	1.904 ± 0.013	0.848 ± 0.009	0.486 ± 0.005	0.183 ± 0.001

System compositions: I—13.75 wt.% PEG-600 − 21.00 wt.% Na/KPB, pH 6.8; II—8.88 wt.% Dextran-64 − 12.86 wt.% Ficoll-400 − 0.01 M NaPB, pH 8.6; III—10.98 wt. % Dextran-64 = 15.95 wt. % Ficoll-400 − 0.24M NaSCN − 0.01 M NaPB, pH 8.6; IV—12.33 wt. % Dextran-64 − 6.05 wt.% PEG-6000 − 0.15M NaCl − 0.01 M NaPB, pH 7.4.

**Table 6:** Partition coefficients, K, for hen egg white lysozyme and its mixtures with different binding partners in the indicated ATPSs (protein/binding partner molecular ratio 1:1 in each case).

Binding Partner	ATPS (K Value)			
	I	II	III	IV
-	2.728 ± 0.010	2.278 ± 0.006	0.890 ± 0.007	1.239 ± 0.008
Glucose	2.645 ± 0.025	2.119 ± 0.035	0.829 ± 0.005	1.206 ± 0.024
Mannose	2.848 ± 0.028	2.311 ± 0.022	0.811 ± 0.005	1.236 ± 0.012
N-Acetyl-D-glucoseamine	2.860 ± 0.035	2.402 ± 0.002	0.818 ± 0.003	1.214 ± 0.010
N,N′-Diacetylchitobiose	2.563 ± 0.032	1.311 ± 0.013	0.826 ± 0.001	1.177 ± 0.009
N,N′,N″-Triacetylchitotriose	2.033 ± 0.052	0.461 ± 0.006	0.803 ± 0.004	1.106 ± 0.007

System compositions: I—12.33 wt.% Dextran-64 − 6.05 wt.% PEG-6000 − 0.15M NaCl − 0.01 M NaPB, pH 7.4; II—13.75 wt.% PEG-600 − 21.00 wt.% Na/KPB, pH 6.8; III—8.88 wt.% Dextran-64 − 12.86 wt.% Ficoll-400 − 0.01 M NaPB, pH 8.6; IV—8.88 wt.% Dextran-64 − 12.86 wt.% Ficoll-400 − 0.25 M Na_2_SO_4_ − 0.01 M NaPB, pH 8.6.

**Table 7:** Signature for three drugs with known and unknown biological activity.

	ATPS				
Drug	A	B	C	D	Activity Level
X	1	0.3	0.6	1	10
Y	0.5	0.6	1	0.7	0
Z (unknown)	0.7	1	0.8	0.9	unknown

## Data Availability

The data are contained within the article.
